# Supplementary material for: Crosstalk between Thyroid Hormone and Corticosteroid Signaling Targets Cell Proliferation in Xenopus tropicalis Tadpole Liver
Source: Int J Mol Sci. 2022 Nov 8;23(22):13715. doi: 10.3390/ijms232213715 (PMC9692397; doi:10.3390/ijms232213715)
Supplement: Supplementary file 1 [file ijms-23-13715-s001.zip › Figure S1.pdf]

Figure S1. Decision tree highliting the effects of the hormonal treatments. n stands for No and y for Yes, t is for threshold.

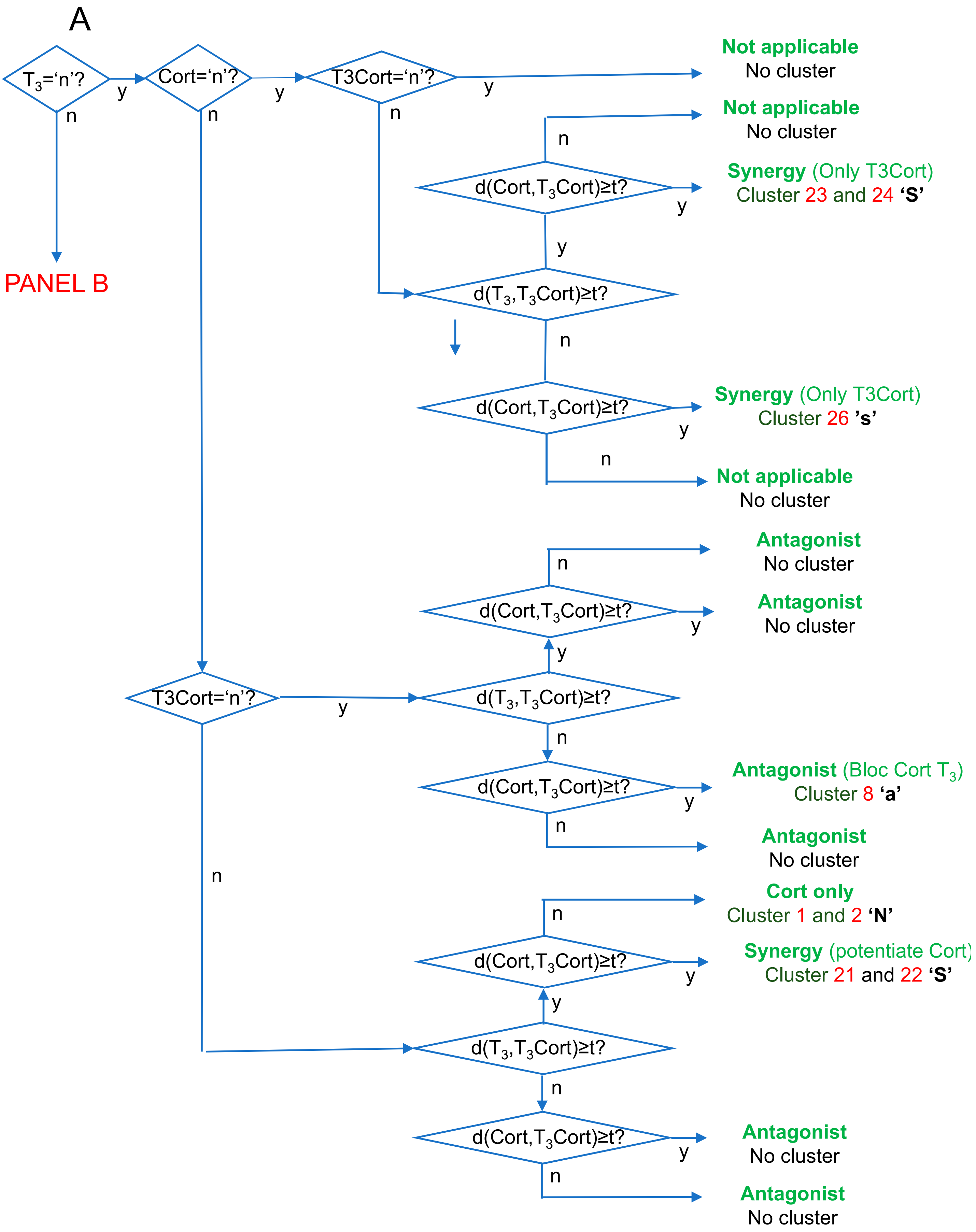

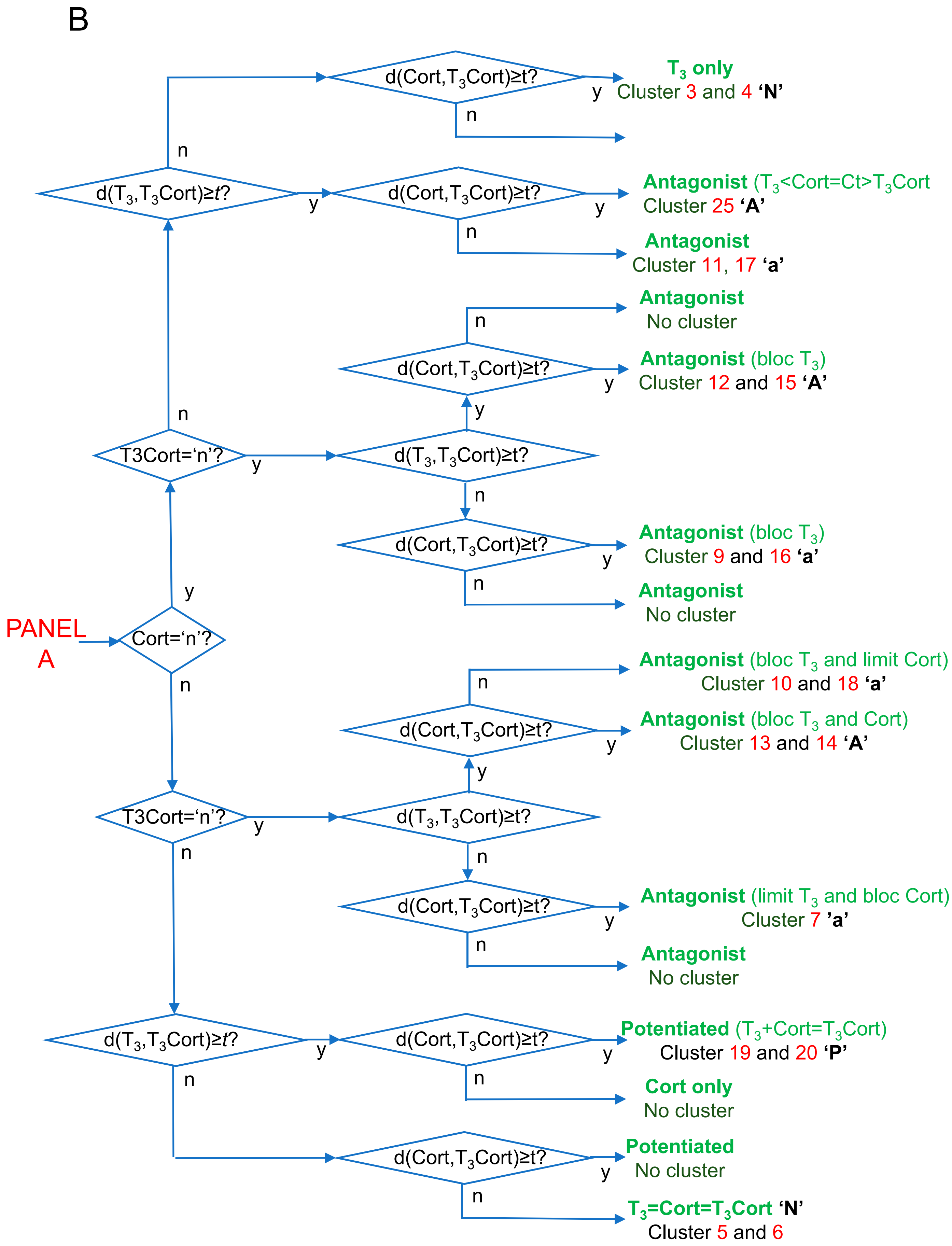
B
PANEL A
Cort='n'?
T3Cort='n'?
d(T3,T3Cort)≥t?
d(Cort,T3Cort)≥t?
y
n
T3 only
Cluster 3 and 4 'N'
Antagonist (T3<Cort=Ct>T3Cort
Cluster 25 'A'
Antagonist
Cluster 11, 17 'a'
Antagonist
No cluster
Antagonist (bloc T3)
Cluster 12 and 15 'A'
Antagonist (bloc T3)
Cluster 9 and 16 'a'
Antagonist
No cluster
Antagonist (bloc T3 and limit Cort)
Cluster 10 and 18 'a'
Antagonist (bloc T3 and Cort)
Cluster 13 and 14 'A'
Antagonist (limit T3 and bloc Cort)
Cluster 7 'a'
Antagonist
No cluster
Potentiated (T3+Cort=T3Cort)
Cluster 19 and 20 'P'
Cort only
No cluster
Potentiated
No cluster
T3=Cort=T3Cort 'N'
Cluster 5 and 6
